# Comparative Nutritional and Histological Analysis of Malabar Red Snapper (*Lutjanus malabaricus*) and Asian Seabass (*Lates calcarifer*)

**DOI:** 10.3390/ani14121803

**Published:** 2024-06-17

**Authors:** Kathiresan Purushothaman, Rachel Ho Jia Wen, Muhammad Hazim bin Mohamed, Saraphina Dianne Tneo Rwei Qing, Lee Heng Wuan, Bing Liang, Nguyen Thanh Vu, Michael Voigtmann, Charles McLean Press, Grace Loo, Saraswathy Bisa, Jose A. Domingos, Dean R. Jerry, Shubha Vij

**Affiliations:** 1 School of Applied Science, Republic Polytechnic, 9 Woodlands Avenue 9, Singapore 738964, Singapore; rachel.ho@jcu.edu.au (R.H.J.W.); hazim.mohamed@jcu.edu.au (M.H.b.M.); saraphina_tneo@rp.edu.sg (S.D.T.R.Q.); lee_heng_wuan@rp.edu.sg (L.H.W.); grace_loo@rp.edu.sg (G.L.); 2 Tropical Futures Institute, James Cook University Singapore, 149 Sims Drive, Singapore 387380, Singapore; bing.liang@my.jcu.edu.au (B.L.); vu.nguyen2@jcu.edu.au (N.T.V.); jose.domingos1@jcu.edu.au (J.A.D.); dean.jerry@jcu.edu.au (D.R.J.); 3 Department of Preclinical Sciences and Pathology, Faculty of Veterinary Medicine, Norwegian University of Life Sciences, 1433 Ås, Norway; charles.press@nmbu.no; 4 Marine Aquaculture Centre, Singapore Food Agency, 52 Jurong Gateway Road, JEM Office Tower, #14-01, Singapore 608550, Singapore; 5 Singapore Aquaculture Technologies (SAT) Pte Ltd., Singapore 308931, Singapore; michael@wintershine.net; 6 Faculty of Biosciences and Aquaculture, Nord University, 8026 Bodø, Norway; bisa.saraswathy@nord.no; 7 ARC Research Hub for Supercharging Tropical Aquaculture through Genetic Solutions, James Cook University, 1 James Cook Drive, Townsville, QLD 4811, Australia

**Keywords:** Malabar red snapper, gastrointestinal micromorphology, goblet cells, fillet fatty acids

## Abstract

**Simple Summary:**

This study presents an analysis of the gastrointestinal tract (GIT) of Malabar red snapper, comparing it with Asian seabass. It highlights slight differences in moisture content, crude protein, and ash between the two species, with Malabar red snapper showing higher essential fatty acid levels. Additionally, Malabar red snapper’s GIT features protective mechanisms in the esophagus and distinct glandular densities in the stomach. The intestine also shows variations in goblet cell distribution and acid mucin secretion along its length. These findings offer valuable insights for the aquaculture sector, especially concerning Malabar red snapper.

**Abstract:**

This study offers a comprehensive morpho-histological analysis of the gastrointestinal tract (GIT) of the Malabar red snapper. A comparison of its GIT morphology with that of the Asian seabass reveals similarities and differences between the two species. Additionally, the moisture content, crude protein, and ash in the fillets of Malabar red snapper and Asian seabass were slightly different, with Malabar red snapper exhibiting higher levels of essential fatty acids. Furthermore, higher levels of the polyunsaturated fatty acid (PUFA)/saturated fatty acid (SFA) ratio and docosahexaenoic acid (DHA)/eicosapentaenoic acid (EPA) ratio, and a lower omega-6/omega-3 ratio, were observed in Malabar red snapper compared to Asian seabass. The Malabar red snapper’s esophagus featured protective mechanisms such as simple columnar epithelial cells, mucous-secreting glands, and goblet cells that were predominantly stained for acid and neutral mucosubstances. Furthermore, its stomach, with mucus cells that were weakly stained for acid mucosubstances, exhibited distinct regions with varying glandular densities, with the pyloric region featuring few glands. The pyloric caeca of the fish were composed of five finger-like structures and few goblet cells. Several goblet cells gradually increased from the anterior to the posterior region of the intestine. These findings provide useful insights for the aquaculture sector, focusing on Malabar red snapper.

## 1. Introduction

Over the past decades, aquaculture has emerged as one of the fastest-growing food-producing sectors, playing a crucial role in meeting the escalating demand for seafood. Worldwide, more than three billion people depend on fish as their daily source of protein, emphasizing the importance of both the aquaculture and fisheries sectors in preventing malnutrition [1]. Malabar red snapper (hereafter referred to as red snapper, *Lutjanus malabaricus*) and Asian seabass (*Lates calcarifer*) are prominently cultured species in Southeast Asia. Red snapper is a highly valued and popular fish that is known for its bright red pigmentation. The Asian seabass, which is also known as barramundi, is an important food fish obtained from both aquaculture and fisheries across Southeast Asia, Australia, North America, and Europe.

Red snapper and Asian seabass are the key cultured food fishes in Singapore [2,3,4]. Farmed red snappers are usually offered pelleted feeds containing more than 40% protein, compared to 45–55% in the feeds of Asian seabass [5]. An analysis of fillet fatty acid profiles by Durmus [6] revealed that all 13 seafood species studied had elevated levels of eicosapentaenoic acid (EPA, C20:5n-3) and docosahexaenoic acid (DHA, C22:6n-3) and had higher levels of Σn-3 poly unsaturated fatty acids (PUFAs) than Σn-6 PUFAs, underscoring the nutritional value of seafood. Other studies that profiled the fatty acids of 8 and 14 food fish species, respectively, yielded similar results [7,8]. It is important to note that a well-functioning gastrointestinal tract (GIT) is pivotal in processing diet-derived nutrients to ensure the sufficient growth of farmed fish and produce high-quality fillets rich in fatty acids that are critical for human health.

The GIT plays a crucial role in the nutrition, growth, and survival of fish across diverse environmental conditions [9,10]. The structure of the GIT varies among fish species, and based on their dietary habits, fish species are categorized into herbivores, carnivores, and omnivores [11,12]. A given species’ dietary preferences can have a bearing on the morphology of its GIT, reflecting its specific nutritional needs. In the wild, red snappers are carnivorous, opportunistic feeders, feeding on other small fishes, shrimps, worms, octopuses, squids, plankton, and zoobenthos [13]. Asian seabass are also carnivorous and opportunistic feeders, and these voracious feeders have a high preference for crustaceans and smaller fishes [14].

Given the intimate connection between diet and structure of the GIT, it is essential to undertake comprehensive analyses of its morphological and histological features. Such an approach has shed light on feeding behaviors [15], habitat preferences [16,17], and diet-caused undesirable changes in the gastrointestinal tract [12,18]. In the present study, we have adopted a multifaceted staining approach, combining various histochemical techniques to reveal the differences in the GIT structures of two key cultured fish species. We have previously published corresponding results for Asian seabass [19]. The aim of this study is to analyze the gastrointestinal tract micromorphology and nutritional profile of Malabar red snapper compared to Asian seabass [19].

## 2. Materials and Methods

### 2.1. Ethics Statement

This study was approved by the Singapore Food Agency (SFA) and Institutional Animal Care and Use Committee, IACUC (approval ID: 2021-A010). All the procedures were executed based on the guidelines of National Advisory Committee on Laboratory Animal Research (NACLAR).

### 2.2. Sampling for Histological Analysis and Nutritional Profiling

Histology-based GIT micromorphology of Asian seabass was previously reported by us [19]. Hence, in the present study, we investigated the histomorphology of the GIT of red snapper only. We have also carried out a comparative study of the results from the present study of red snapper with those from our previous study on Asian seabass.

In total, 10 live red snappers (4 months old) were purchased from Prime Supermarket, Singapore. The purchased fish were euthanized using 2% tricaine before recording their body weight (BW), and total and standard length (TL & SL). The fish were then dissected, and the lengths of their intestines (IL) were measured. Furthermore, intestine samples were also collected from a total of 6 fish for the histology study. The intestinal coefficient (IC) was determined using the formula IC = IL/SL.

**Figure 1:**
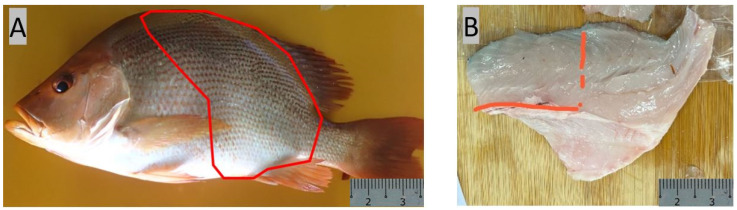
Section of Malabar red snapper fillet that was collected for fatty acid, moisture, protein, and ash analysis. (**A**) Fish, (**B**) fillet.

For nutritional profile analysis, the fish (red snapper and Asian seabass, 10 each) were sourced from a local fish farm. Both groups of fish were fed with the same commercial feed containing 44% protein (Uni-President, Dĩ An, Vietnam). A total of six fish from each species (of similar body weight) were euthanized with 2% tricaine. Subsequently, muscle samples adjacent to the dorsal and tail fin were collected (Figure 1), snap-frozen in liquid nitrogen, and stored at −80 °C for downstream analysis.

### 2.3. Histological Analysis

The collected intestine samples were preserved in 10% buffered formalin for 48 h. Post-fixation, a systematic dehydration process was initiated using ethanol, with a gradient concentration ranging from 50% to 100%. The specimens were then embedded in paraffin wax [20,21,22,23,24]. Two cross sections of approximately 4–5 µm in thickness were mounted onto slides and incubated at 37 °C for 24 h. A sequential dewaxing using xylene and rehydration using alcohol was performed to prepare the specimens for further analysis. The sections were stained with hematoxylin-eosin (HE), with both Alcian Blue (AB, pH 2.5) and periodic acid-Schiff (PAS), for the detection of neutral and acidic glycoproteins [19]. Transverse sections of each part of the GIT (esophagus, stomach, pyloric caeca, intestine) were examined. Microphotography of each slide (individual parts of the GIT) was conducted using a Leica ICC50 HD camera attached to a Leica DM500 microscope (Leica Microsystems, Wetzlar, Germany). The AB-PAS-stained goblet cells were counted using Fiji Software version 2.3.1 [24,25].

### 2.4. Proximate Composition of Fish Fillet and Feed

#### 2.4.1. Fillet Fatty Acid Extraction and Analysis

Fatty acids in the fillet were determined at the facilities of Sustainable Technology & Analytical Research (STAR) Laboratory, Republic Polytechnic, Singapore, employing the method outlined by O’Fallon et al. [26]. Fifteen grams of fillet from each sample were placed in a freeze dryer for a week. These samples were then ground for 10–15 s to determine their moisture content. The obtained powder (0.5 g) was transferred to a 15 mL Falcon tube, and then C13:0 internal standard (1.0 mL, 0.5 mg C13:0/mL in methanol), 10 N KOH in water (0.7 mL), and methanol (5.3 mL) were added to the samples. The tubes were then incubated at 55 °C for 1.5 h, with intermittent shaking by hand for 5 s every 20 min to allow efficient permeation, dissolution, and hydrolysis. After cooling, 24 N H_2_SO_4_ in water (0.58 mL) was added, and the tube was inverted several times to mix the contents in the tube thoroughly. A second incubation at 55 °C for 1.5 h was conducted with K_2_SO_4_. After the synthesis of fatty acid methyl esters (FAME), hexane (3 mL) was added, followed by 5 min of mixing with a vortex and 5 min of centrifugation. The resulting hexane layer, containing FAME, was transferred to a gas chromatography (GC) vial, sealed, and stored at −20 °C, to ensure the stability of samples for subsequent GC analysis. Capillary gas chromatography on a SP2560, 100 m × 0.25 mm × 0.20 μm capillary gas column (Sigma-Aldrich) was used for this study. The system was equipped with a flame ionization detector (250 °C) and split injector (250 °C). Oven temperature program of the system was set to isothermal at 140 °C (5 min), ramped at 40 °C/min to 240 °C (hold 25 min). Hydrogen served as the carrier gas (flow rate, 1.12 mL/min; linear velocity, 20 cm/s; split ratio 15:1) and fatty acids were identified by comparing their retention times with SupelcoTM 37 component FAME Mix standards (47885-U). Individual fatty acids were quantified as area percentages of the total fatty acids.

#### 2.4.2. Fillet Moisture, Protein and Ash Analyses

Crude protein was analysed using the Kjeldahl method (wet basis), wherein the nitrogen content of the fish muscles was determined and converted to total crude protein by multiplying the nitrogen content by a conversion factor of 6.25 [27]. Analysis of the moisture and ash of individual fish muscles was conducted using the gravimetric method. This process included heating a 2 g sample at 105 °C overnight in the oven (DAIHAN ThermoStable™, DKSH, Seoul, Republic of Korea), followed by a subsequent 4 h heating in a muffle furnace (Nabertherm, LE 14/11, Bremen, Germany) at 600 °C.

#### 2.4.3. Feed Crude Fat, Moisture, Protein and Ash Analyses

Crude fat, crude protein, ash, and moisture contents of the feed were analyzed. Crude fat was extracted using hexane, and the content was measured by acid hydrolysis [28]. Crude protein was determined using the Dumas method, wherein the nitrogen content of the fish feed was measured and converted to total crude protein by multiplying the nitrogen content by a conversion factor of 6.25 [27]. Ash and moisture contents were determined using the gravimetric method. For ash analysis, the fish feed was burned by igniting the sample at 550 °C in an electric furnace [28]. For moisture analysis, the fish feed was dried in a vacuum oven at 100 °C from 5 to 5.5 h [28].

### 2.5. Statistical Analysis

We utilized R (version 4.3.3) packages for statistical analysis and the codes were run on RStudio (2024.04.1 Build 748). Kruskal–Wallis test followed by Dunn’s test was used to compare the number of goblet cells in different regions of the gastrointestinal tract as the data did not satisfy the assumption of the test. In addition, the differences in the fillet proximate composition and fatty acids in red snapper and Asian seabass were checked using two-sided *t*-test with a significance threshold of *p*-value < 0.05. All the assumptions were checked before carrying out the *t*-test. Transformations were done wherever necessary, and the non-parametric equivalent of the *t*-test was chosen for analysis of non-parametric data. All the data in this study are presented as mean ± SD.

## 3. Results

### 3.1. Morpho-Histological Analysis of the Gastrointestinal Tract of Red Snapper

The body weight (BW), standard length (SL), total length (TL), intestinal length (IL), and calculated intestinal coefficient (IC) of the two groups of fish are shown in Table 1. The calculated IC of Asian seabass was slightly lower than that of red snapper [19].

**Figure 2:**
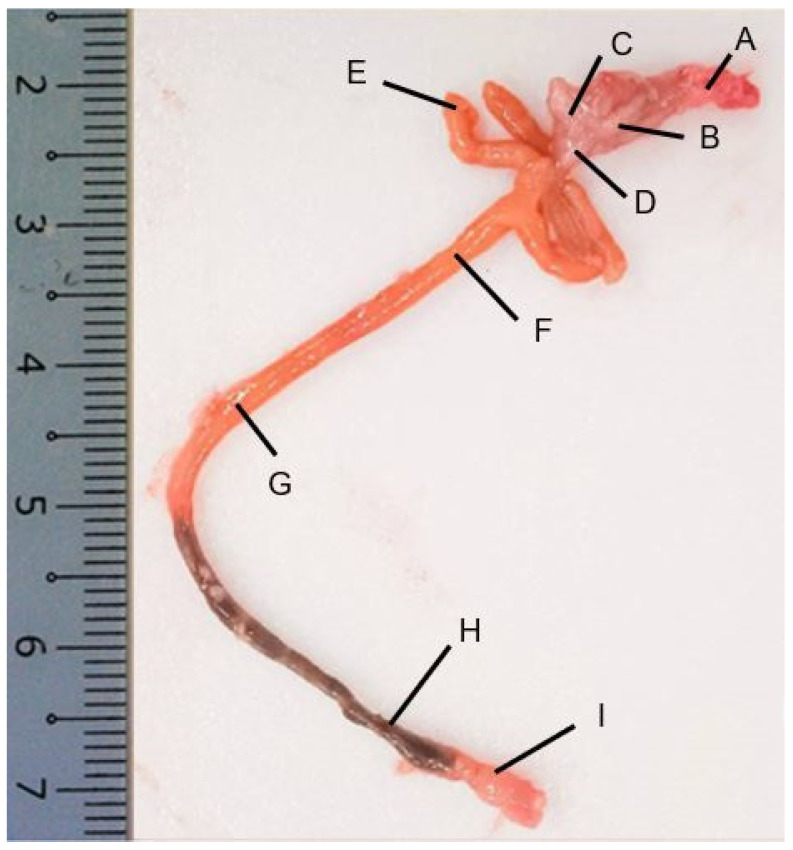
Morphology of the red snapper gut. (A) Esophagus, (B) cardiac stomach, (C) fundic stomach, (D) pyloric stomach, (E) pyloric caeca, (F) anterior intestine, (G) mid intestine, (H) posterior intestine, and (I) rectum.

**Figure 3:**
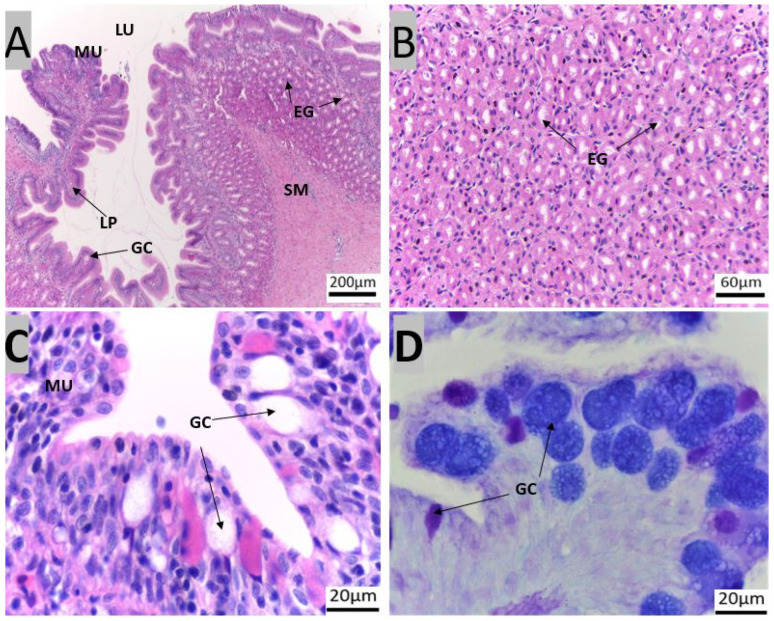
Transverse sections of red snapper esophagus. The tissues were stained with Hematoxylin and Eosin (H&E) ((**A**): 10×, (**B**): 40×, (**C**): 100×), and subsequently stained with Alcian Blue-Periodic acid-Schiff (AB-PAS) ((**D**): 100×). Abbreviations: Esophageal glands (EG), goblet cells (GC), lamina propria (LP), lumen (LU), mucosa (MU), sub-mucosa (SM).

The GIT of red snapper consists of the esophagus, which is connected to the stomach, followed by the pyloric caeca, the intestine, and finally the rectum (Figure 2). Four layers could be recognized in the esophagus, mucosa, sub-mucosa, muscularis, and serosa (Supplementary Figure S1). The esophageal mucosa exhibited a folded epithelial structure, featuring a simple columnar epithelium with numerous goblet cells (Figure 3A). Esophageal glands were evident within the submucosal region (Figure 3A,B). The goblet cells were predominantly stained purple (stained for both PAS magenta and AB blue = purple) and blue (stained only for AB = blue, Figure 3D).

**Figure 4:**
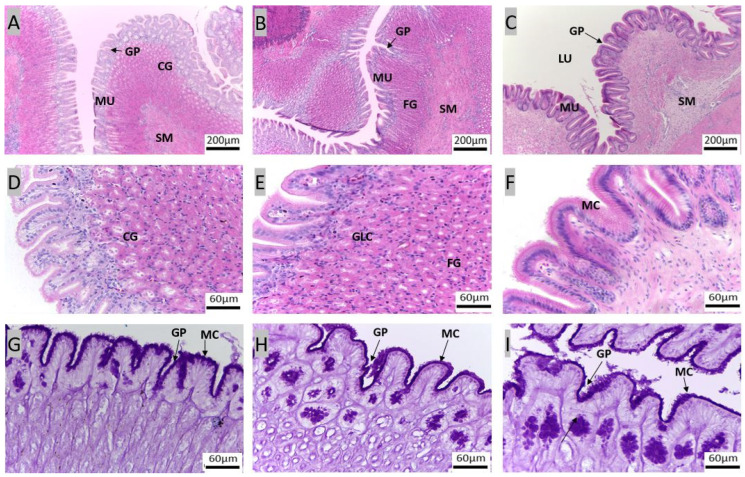
Transverse sections of red snapper stomach. Cardiac stomach ((**A**): 10×, (**D**): 40×), fundic stomach ((**B**): 10× and (**E**): 40×), and pyloric stomach ((**C**): 10× and (**F**): 40×). The sections are stained with H&E. Cardiac stomach ((**G**): 100×), fundic stomach ((**H**): 100×), and pyloric stomach ((**I**): 100×) stained with AB- PAS. Abbreviations: cardiac gland (CG), fundic gland (FG), gastric pits (GP), lumen (LU), mucosa (MU), submucosa (SM), mucosal cells (MC), gland cells (GLC).

The stomach exhibited a sac-like morphology with a surface characterized by secretory simple columnar epithelium. The stomach is divided into three parts: cardiac, fundic, and pyloric regions (Figure 4, Supplementary Figure S2). A thick mucosa with few glands at the bottom of the gastric pits was characteristic of the pyloric region of the stomach. Predominantly, the cardiac and fundic regions harbor gastric glands, identified as cardiac and fundic glands, respectively (Figure 4A–E). All the regions in the stomach also contained mucus-secreting cells near the luminal part of the stomach. Notably, the surface mucus cells exhibited strong staining for PAS and weak staining for AB (pH 2.5, Figure 4G–I).

**Figure 5:**
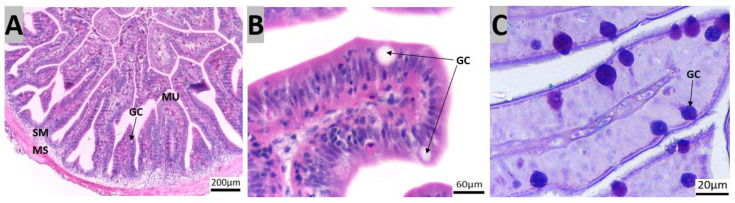
Transverse sections of red snapper pyloric caeca. The sections are stained with H&E ((**A**): 10× and (**B**): 100×) and PAS-AB ((**C**): 100×). Abbreviations: goblet cells (GC), mucosa (MU), muscularis (MS), submucosa (SM).

The pyloric caeca were observed as five finger-like structures at the end of the pylorus of the stomach (marked E in Figure 2). The mucosa of the pyloric caeca was characterized by lengthy folds, and consisted of a layer of simple columnar epithelium, which included absorptive cells and cylindrical goblet cells. Below the epithelium we noted lamina propria with loose connective tissue (Figure 5A,B). The pyloric caeca had thin mucosal folds, and this region had a sparser population of goblet cells compared with other regions of the intestine. The pyloric caeca showed positive PAS and AB staining (Figure 5C).

**Figure 6:**
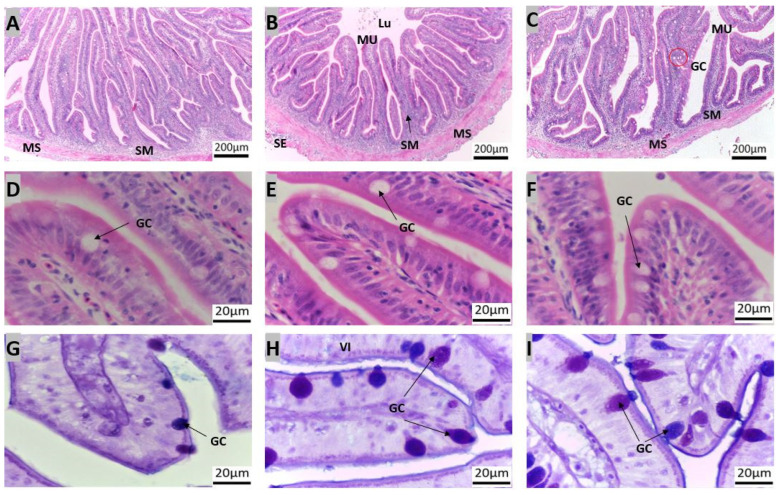
Transverse sections of red snapper anterior intestine. The sections are stained with H&E ((**A**): 10×, (**D**): 100×) and AB-PAS ((**G**): 100×), mid intestine H&E ((**B**): 10× and (**E**): 100×) and PAS-AB ((**H**): 100×), and posterior intestine H&E ((**C**): 10× and (**F**): 100×) and PAS-AB ((**I**): 100×). Abbreviations: goblet cells (GC), lumen (LU), muscularis propria (MS), serosa (SE), submucosa (SM), Vili (VI).

The intestine present between the pyloric caeca and the rectum (Figure 2, marked F–H) was segmented into the anterior, middle, and posterior regions. The simple columnar epithelium consisted of absorptive and goblet cells, with a predominance of the goblet cell type, which stained positive for both AB and PAS (Figure 6G–I).

The posterior intestine was characterized by shorter transverse mucosal folds and numerous goblet cells (Figure 6). These segments exhibited mucosal folds, with a clear distinction between the lamina propria and the submucosa. Towards the posterior intestine, goblet cell counts increased significantly. The length of the microscopic mucosa fold decreased from the anterior to posterior intestine while the thickness increased (Figure 6A–F). Goblet cell abundance increased from anterior to posterior, with a higher prevalence of AB-positive cells than PAS-positive cells (Table 2).

**Figure 7:**
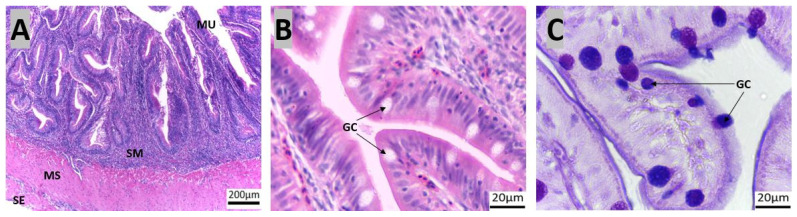
Transverse sections of the red snapper rectum. The sections are stained with H&E ((**A**): 10×, (**B**): 100×) and PAS-AB, ((**C**): 100×). Abbreviations: goblet cells (GC), mucosa (MU), muscularis propria (MS), serosa (SE), and submucosa (SM).

The rectum extended ventrally from the posterior intestine (Figure 2, marked I) and terminated near the anal fin. The rectum, similar to the intestine, had several mucosal folds without glands (Figure 7A,B). Goblet cell counts increased progressively along the intestinal tract and the goblet cells showed a higher prevalence of AB-positive cells than PAS-positive cells (Table 2; Figure 7C).

### 3.2. Nutritional Profile Analysis

The numbers of saturated fatty acids (∑SFA) and total polyunsaturated fatty acids (∑PUFA) are slightly higher in red snapper than in Asian seabass. On the other hand, the total numbers of monounsaturated fatty acids (∑MUFA) are higher in Asian seabass. Specifically, the content of the majority of fatty acids within these three groups in the Malabar red snapper were significantly higher than those in Asian seabass (Table 3). The results of the proximate analysis of fish feed are shown in Supplementary Table S1.

Importantly, the amounts of Methyl alpha-linolenic acid (C18:3n3), Methyllinoleic acid (C18:2n6c), Methyl *cis*-11,14-eicosadienoic acid (C20:2), Methyl *cis*-11,14,17-eicosatrienoic acid (C20:3n3), and Methyl *cis*-13, 16-docosadienoic acid C22:2 were significantly higher in Malabar red snapper. On the other hand, the amounts of Methyl gamma-linolenic acid (C18:3n6), Methyl *cis*-8,11,14-eicosatrienoic acid (C20:3n6), and Methyl *cis*-5, 8, 11, 14-eicosatetraenoic acid (C20:4n6) were significantly higher in Asian seabass. Additionally, the amounts of essential fatty acid linoleic acid (18:2n6t), *cis*-5, 8, 11, 14, 17-eicosapentaenoic acid (C20:5n-3), and Methyl *cis*-4, 7, 10, 13, 16,19-docosahexaenoic acid (C22:6n-3) were notably higher in the red snapper, although this difference was not statistically significant.

Red snapper showed a higher PUFA/SFA ratio (1.1) compared to Asian seabass (1.0). Red snapper also displayed a lower omega-6/omega-3 ratio (1.1) compared to Asian seabass (1.3). The levels of both DHA and EPA observed in red snapper (13.4% and 3%, respectively) were higher than those of Asian seabass (11.2% and 2.8%, respectively). In addition, the DHA/EPA ratio was also higher in red snapper (4.5) compared to Asian seabass (4.0).

A comparison of total crude protein levels in the two fishes indicated a slightly higher crude protein level in red snapper compared to Asian seabass which was not statistically significant based on a two-tailed *t*-test. The ash contents in red snapper were found to be significantly higher than those of Asian seabass. No significant difference was observed in moisture content (Table 3).

## 4. Discussion

In the natural environment, fish consume a wide variety of prey available at different water depths, reflecting their diverse dietary requirements [29]. The gastrointestinal structure of fish, including their morphology and microstructures, varies with their feeding habits. Several studies have highlighted the direct correlation between these anatomical features and the diet of fish [30]. Consequently, carnivorous fish like red snapper and opportunistic feeders like Asian seabass require specifically tailored feed ingredients to maintain the optimal functionality of their gastrointestinal tract. The selective absorption of dietary nutrients, facilitated by appropriate transporters, promotes the deposition of macronutrients such as protein and lipids in their fillets. Therefore, the GIT is instrumental in the accumulation of proteins and lipids, the determinants of the nutritional quality of fillets. A comprehensive analysis of the micromorphology of the GIT can offer insights into the dietary requirements of fish [31]. In the present study, we provide information about the micromorphology of the GIT as well as the proximate composition and fatty acid composition of the fillet of red snapper. Our study confirmed the carnivorous nature of red snapper, based on the length of the intestine. The esophagus and stomach contained glands and goblet cells while the pyloric caecum, intestine, and rectum were populated by absorptive cells and goblet cells, characterized by neutral and acid mucins. As for the red snapper fillet fatty acids profile, the ratios of PUFA/SFA as well as DHA/EPA were higher compared to Asian seabass. In addition, the omega-6/omega-3 ratio was lower in the case of red snapper.

### 4.1. Protective Components of the Gastrointestinal Tract of Red Snapper

The red snapper has a short esophagus with thick walls, a characteristic well-suited for predatory feeding [32]. The simple columnar epithelium lined with numerous goblet cells observed in the present study has pivotal functions, such as lubrication and hydration to safeguard the integrity of esophageal lining [19,33,34]. In the case of European Seabass (*Dicentrarchus labrax* L.), common dentex (*Dentex dentex*, *Pisces*, *Sparidae*), and gilthead seabream *(Sparus aurata* L.), the esophagus contains a stratified columnar epithelium with numerous goblet cells [35,36,37]. On the other hand, the esophagus of Atlantic salmon was reported to have a stratified squamous epithelium with scattered goblet cells [38]. In humans, the simple columnar epithelium in the esophagus is replaced by a squamous epithelium [39]. There are specific molecules that are drivers of this differentiation, as shown in a study using P63 knockdown zebrafish [40]. Regarding the esophageal goblet cells, some studies have suggested that they produce mucosubstances including mucins (glycosylated proteins) and mucopolysaccharides (glycosaminoglycans) [41]. Of these, mucins within the epithelium lubricate food, act as a barrier or substrate for selected microbes, and take part in immune defense. On the other hand, the mucopolysaccharides provide structural support and hydration [41]. Similar to the observation in the present study, the esophagi of seabream, seabass, and common dentex contain both acidic and neutral mucosubstances [35,36,37]. The mucous-secreting cells in the simple columnar epithelium work along with esophageal glands to safeguard the esophageal lining [42]. The esophageal glands were identified in both red snapper and Asian seabass [19]. Thus, the protection in the esophagus is provided by a lining of either simple/stratified columnar or stratified squamous epithelial cells in different types of fish and works in concert with goblet cells and glands [43].

### 4.2. Components of the Gastrointestinal Tract Crucial for Digestion and Nutrient Absorption

The stomach plays a role in the storage and digestion of food [44]. Some fish species, such as zebrafish (*Danio rerio*), ballan wrasse (*Labrus bergylta*), garfish (*Belone belone*), and needlefish (*Tylosurus gavialoides* and *Strongylura leiura ferox*) do not have stomachs [45,46,47]. The morpho-histological results of the red snapper stomach in this study are comparable to our previous study on Asian seabass [19]. The stomach of the gilthead seabream is lined by cuboidal epithelium, while the stomachs of Atlantic salmon and European seabass are lined by columnar epithelium [35,36,37,48]. The structure of the stomach, the gland distribution, and the types of mucosubstances of red snapper are observed to be similar to those in Asian seabass [19]. The pyloric stomach’s fewer glands indicate a primary role of food retention rather than digestion. This storage function, aided by the pyloric sphincter, allows for extended digestion time, observed in fishes like the walking catfish and red-bellied piranha [12]. Some fish species exhibit a pyloric stomach that lacks glands [33,49,50]. The stomach of red snapper was stained magenta with AB-PAS, indicating the presence of neutral glycoproteins and the secretion of other neutral mucosubstances. Mucins and bicarbonate act together to create a neutral pH in rat stomach [51]. Similar mechanisms may also exist in fishes [19,33,36,52]. The epithelia in the stomach of common dentex, European seabass, Atlantic salmon, and seabream contain neutral mucosubstances including neutral glycoproteins [36,37,48]. Comparative analysis of the morphology and micromorphology of the stomach of red snapper with that of Asian seabass and other fish species underscores similarities and differences in gland distribution, epithelial type, mucosubstance composition, and functional roles, providing insights into their adaptation strategies for food storage and digestion.

The pyloric caeca are observed in approximately 60% of teleosts, with a heightened prevalence that is particularly notable among carnivorous fish species. The pyloric caeca play a significant role as an adaptive mechanism to enhance the surface area of the fish gut for increased nutrient uptake and absorption without increasing intestinal length [33,53]. Atlantic salmon typically possess from 55 to 75 pyloric caeca, common dentex generally has from 3 to 6, European seabass typically has from 4 to 5, and Atlantic cod (*Gadus morhua*) commonly has ∼700 [35,37,48,54]. These structures play a crucial role in digestion and nutrient absorption. The pyloric caeca of European seabass contain neutral mucin, while those of Atlantic salmon and common dentex produce both acidic and neutral mucins [35,37,52]. Both red snapper and Asian seabass exhibited pyloric caeca characterized by five finger-like projections. Regarding acidic and neutral glycoproteins, both were detected in red snapper, but Asian seabass contained only neutral glycoproteins, which aid in lubricating the epithelium, shielding it from physical and chemical damage, as well as pathogens present in the gut lumen [41]. Comparing the pyloric caeca and mucin composition between red snapper, Asian seabass, and other fish reveals species-specific digestive adaptations.

In most teleost fishes, the intestine wall of the GIT is a three-layered structure which consists of mucosa, muscularis, and serosa [45]. It is widely known that the intestine length and intestinal coefficient of teleost fishes can provide an insight into their feeding pattern and habits [19]. In general, fish with a higher IC are classified as herbivores (0.8–15), fish with a lower IC are classified as carnivores (0.2–2.5), and those that fall between that range are omnivore (0.6–8.0) [49,55,56,57,58]. Our study shows that the Asian seabass and red snapper both have relatively low ICs of 1.1 ± 0.04 cm and 1.29 ± 0.17 cm, respectively, confirming their classification as predominantly carnivorous species. The abundance of both acidic and neutral mucosubstances throughout the intestine suggests their involvement in lubrication and defence in this organ [59,60,61]. There is a progressive increase in goblet cells along the intestine (from anterior to posterior) as shown by the histological staining (Figure 6). Similarly, the number of goblet cells increased from the pyloric caeca to the rectum of Atlantic salmon, as reported by Sørensen et al. [52]. These segments of Atlantic salmon have a higher proportion of acid mucins compared to neutral mucins [52]. Nevertheless, the pH of the intestinal chyme in Atlantic salmon was reported to be around 8.1 [62]. Our previous study on Asian seabass also pointed to a similar trend in the increase in goblet cells and the presence of mucin types. A higher number of goblet cells likely enhances mucus production to protect the intestinal lining and facilitate fecal expulsion. Conversely, the number of goblet cells decreased from the anterior intestine to the rectum of zebrafish [63]. As for the thickness of intestinal folds in red snapper, we observed a progressive increase from the anterior to the posterior section. The results on goblet cell distribution as well as the villi height corroborate with the findings of other fishes [19,59,64]. Both the red snapper and Asian seabass are carnivores and usually have more protein and less fiber in their diet. Fiber and plant matter encourage intestinal peristalsis, while fiber deficiency necessitates a thicker and stronger muscularis externa in the rectum compared to the intestine and pyloric caeca [16,65]. Acidic glycoproteins would function more as a lubricative and protective secretion. Therefore, the increase in the number of goblet cells from the intestine to the rectum is likely indicating the need for more lubrication in these segments of the GIT. The examination of intestinal characteristics, including its length, goblet cell distribution, and mucin composition, across carnivorous fish species like red snapper and Asian seabass, highlights adaptations for efficient protection.

### 4.3. Fatty Acid Profile in the Fillet of Red Snapper

Fatty acids play a significant role in the physiological processes of organisms. Although not categorized as “essential”, SFAs at moderate levels have pivotal roles in maintaining cell membrane integrity and hormone synthesis. Palmitic acid (C16:0) was the dominant SFA in both red snapper and Asian seabass. Abbas et al. [66,67] and Durmus [6] also reported the dominance of palmitic acid among other SFAs in fishes. High concentrations of MUFA in fish fillets can point to the consumption of zooplankton and copepods [68]. The *Clupea harengus pallasi* (Pacific herring) also had higher MUFA and SFA content (48.12% and 26.21%, respectively) in its fillet [69]. In the aforementioned fishes and red snapper, oleic acid was the MUFA with the highest content; 20% for sea bream and 23.8% for Pacific herring. As for the SFAs, palmitic acid was the major constituent in this category; 16.1% for seabream and 18.4% for Pacific herring [7,69].

Red Snapper had a higher SFA and MUFA content compared to other fish species such as *Merlangius merlangus* (Whitting; 29.6% and 19.2%, respectively), *Cyprinus carpio* (common carp; 28% and 13.8%, respectively), and *Sardinops sagax* (sardine; 25.2% and 14.2% respectively) [7,69].

Polyunsaturated fatty acids (PUFA) are essential components of cell membranes, specifically within the phospholipids. Omega-3 (n-3) long-chain (LC) PUFAs like eicosapentaenoic acid (EPA; 20:5n-3) and docosahexaenoic acid (DHA; 22:6n-3) are regarded as healthy fatty acids. These two fatty acids are vital for synthesizing specific eicosanoids such as prostaglandins, thromboxanes, and leukotrienes. In humans, the consumption of PUFAs has been long recognized to bestow numerous health benefits. DHA is an important component of the brain and vision while EPA is crucial for the prevention of cardiovascular diseases, cognitive disorders, and numerous cancers [6,70,71].

Our study shows that red snapper has a higher PUFA content compared to the Asian seabass. The EPA and DHA contents are also much higher in red snapper than in Asian seabass. The fatty acid profile showed that Atlantic salmon products from Scotland can contain around 18% EPA+DHA (% of total fatty acids), whereas wild Atlantic salmon contained 21.14 (% of total fatty acids; 1.2 g/100g) compared to 7.58% (1.4 g/100g) in the case of farmed salmon from Norway [72]. Malabar red snapper muscles hold approximately 16% EPA+DHA (% of fatty acids) [73]. Red snapper showed a higher PUFA content when compared to fish species such as bogue (*Boops boops*; 27.5%), mullet (*Mugil cephalus*; 24.8%), sardine (*Sardinella aurita*; 31%), pandora (*Pagellus erythrinus*; 32%), and common sole (*Solea solea*; 33.6%). On the other hand, red snapper showed a lower PUFA than scad (*Trachurus mediterraneous*; 46.4%), red scorpion fish (*Scorpaena scrofa*; 41.6%), and turbot (*Scophthalmus maeoticus*; 41.1%) [7].

The Nordic Nutrition Recommendations indicate that n-3 fatty acids (EPA and DHA) from seafood can reduce the risk of cardiovascular diseases [74]. The EPA and DHA values in Asian seabass and red snapper can be beneficial for humans. This suggests that the consumption of red snapper is also beneficial as it will provide a slightly higher content of PUFA for humans.

Ash content, reflecting the overall mineral levels in tissue, correlates with body development and growth [75]. Red snappers were found to have a higher ash content compared to Asian seabass, pointing to a higher mineral composition, including magnesium, calcium, potassium, and zinc in the former [75,76,77].

## 5. Conclusions

In conclusion, this study provides novel insights into the morphological and histological characteristics of the alimentary canal of red snapper and compared them with those of Asian seabass. The esophageal glands, gastric gland distributions, and intestinal morphology, as well as the mucosubstance compositions of red snapper and Asian seabass, exhibit certain similarities to those of Atlantic salmon, seabass, and common dentex. However, the type of esophageal and stomach epithelium of other fish does not resemble that of red snapper and Asian seabass. The gastrointestinal tract (GIT) of red snapper, characterized by specialized mucosal structures and varying goblet cell distributions, reflects its carnivorous diet and effective nutrient absorption. Comparative analysis with Asian seabass highlighted differences in fatty acid composition, with red snapper showing higher PUFA, PUFA/SFA, and DHA/EPA ratios, and a lower omega-6/omega-3 ratio. These findings enhance our understanding of red snapper’s dietary adaptations and nutritional quality, and can have implications for aquaculture practices, dietary management, and human nutrition.

## Figures and Tables

**Table 1:** Growth parameters of red snapper and Asian seabass.

Species	Body Weight (BW) g	Standard Length (SL) cm	Total Length (TL) cm	Intestinal Length (IL) cm	Intestinal Coefficient (IC) cm
Red Snapper	52.09 ± 6.45	12.00 ± 0.73	14.07 ± 0.92	15.41 ± 2.27	1.29 ± 0.17
Asian Seabass	15.30 ± 3.30	7.80 ± 0.86	9.60 ± 1.00	8.90 ± 0.90	1.14 ± 0.04

Values are presented as mean ± SD. Sample size = 6.

**Table 2:** Goblet cell counts in the intestine and rectum of red snapper.

Organ	Region	Goblet Cell Number
Intestine	Anterior	197 ± 15 ^a^
	Mid	254 ± 16 ^b^
	Posterior	334 ± 30 ^c^
Rectum		507 ± 24 ^d^

The values are mean ± SD. Number of fish is six. Different superscripts indicate significant differences between the intestinal segments.

**Table 3:** Proximate composition and fatty acid composition of red snapper and Asian seabass fillets.

	RS	ASB	RS	ASB
Nutrient Composition	Mean	Mean	SD	SD
% in dry matter				
Moisture	6.80	8.83	3.74	0.60
Crude protein	24.6	23.5	0.01	0.01
Ash ^R^	6.23	4.92	0.26	0.42
Fatty acid composition (% of total fatty acids)				
Methyl butyric acid C4:0 ^R^	0.03	0.00	0.02	0.00
Methyl hexanoic acid C6:0 ^A^	0.06	0.09	0.01	0.01
Methyl octanoic acid C8:0 ^A^	0.02	0.03	0.01	0.00
Methyl lauric acid C12:0 ^A^	0.16	0.33	0.02	0.04
Methyl tridecanoic C13 ^R^	0.01	0.00	0.01	0.00
Methyl myristic acid C14:0 ^R^	2.24	1.72	0.22	0.26
Methyl pentadecanoic acid C15:0 ^R^	0.24	0.20	0.02	0.02
Methyl palmitic acid C16:0	23.03	22.88	0.47	0.59
Methyl heptadecanoic acid C17:0 ^R^	0.33	0.26	0.02	0.02
Methyl stearic acid C18:0	8.55	9.33	0.51	0.52
Methyl arachidic acid C20:0 ^R^	0.19	0.15	0.02	0.01
Methyl heneicosanoic acid C21:0 ^R^	0.05	0.01	0.01	0.01
Methyl behenic acid C22:0	0.14	0.14	0.01	0.01
Methyl lignoceric acid C24:0 ^R^	0.12	0.04	0.04	0.01
ƩSFA	35.19	35.17	1.38	1.51
Methyl myristoleic acid C14:1 ^R^	0.06	0.02	0.01	0.01
Methyl *cis*-10 pentadecenoic acid C15:1 ^A^	0.01	0.02	0.00	0.01
Methyl palmitoleic acid C16:1 ^R^	2.92	2.43	0.26	0.32
Methyl *cis*-10 heptadecenoic acid C17:1 ^A^	0.04	0.19	0.05	0.00
Methyl *trans*-9 eladic acid C18:1n9t	0.36	0.40	0.04	0.02
Methyl *cis*-9 oleic acid C18:1n9c ^A^	21.76	24.40	0.93	1.22
Methyl *cis*-11-eicosenoic acid C20:1n9 ^R^	0.32	0.17	0.03	0.03
Methyl erucic acid C22:1n9 ^R^	0.11	0.05	0.01	0.01
Methyl nervonic acid C24:1n9	0.39	0.18	0.01	0.08
ƩMUFA	25.96	27.86	1.33	1.70
Methyl linolelaidic acid C18:2n6t	0.03	0.03	0.01	0.01
Methyl linoleic acid C18:2n6c ^R^	15.25	13.10	0.35	1.13
Methyl gamma-linolenic acid C18:3n6 ^A^	0.22	0.55	0.02	0.12
Methyl alpha-linolenic acid C18:3n3 ^R^	0.93	0.76	0.07	0.09
Methyl *cis*-11,14-eicosadienoic acid C20:2 ^R^	0.75	0.53	0.03	0.04
Methyl *cis*-8,11,14-eicosatrienoic acid C20:3n6 ^A^	0.38	0.78	0.02	0.18
Methyl *cis*-11,14,17-eicosatrienoic acid C20:3n3 ^R^	0.05	0.01	0.02	0.01
Methyl *cis*-5, 8, 11, 14-eicosatetraenoic acid C20:4n6 ^A^	2.74	3.68	0.29	0.49
Methyl *cis*-13, 16- docosadienoic acid C22:2 ^R^	0.26	0.19	0.02	0.02
Methyl *cis*-5, 8, 11, 14, 17-eicosapentaenoic acid C20:5n3	2.97	2.80	0.15	0.29
Methyl *cis*-4, 7, 10, 13, 16,19-docosahexaenoic acid C22:6n3	13.42	11.24	1.27	1.65
ƩPUFA	36.98	33.67	2.24	4.04

Significant differences (P_adj_ < 0.05) are indicated by superscripts: A for values significantly higher in Asian seabass and R for values significantly higher in red snapper. Saturated fatty acids (SFA), monounsaturated fatty acids (MUFA), and polyunsaturated fatty acids (PUFA). (RS) stands for red snapper, and (ASB) stands for Asian seabass.

## Data Availability

The original contributions presented in the study are included in the article/Supplementary Material, further inquiries can be directed to the corresponding author/s.

## Supplementary Materials

The following supporting information can be downloaded at: https://www.mdpi.com/article/10.3390/ani14121803/s1. Figure S1. Transverse sections of red snapper esophagus. The tissues were stained with H&E, (10×). Abbreviations: Mucosa (MU), sub-mucosa (SM), muscularis (Yellow line, M), serosa (Red line, S), Figure S2. Transverse sections of red snapper stomach. Cardiac stomach (A: 10×), Fundic Stomach (B: 10×), and Pyloric Stomach (C: 10×). The sections are stained with H&E. Abbreviations: Mucosa (MU), sub-mucosa (SM), muscularis (Yellow line, M), serosa (Red line, S). Table S1. Proximate composition and fatty acid composition of the feed bed both Red Snapper and Asian Seabass.
