# Supplementary figures and images for: Comparative Nutritional and Histological Analysis of Malabar Red Snapper (Lutjanus malabaricus) and Asian Seabass (Lates calcarifer)
Source: Animals (Basel). 2024 Jun 17;14(12):1803. doi: 10.3390/ani14121803 (PMC11200453; doi:10.3390/ani14121803)

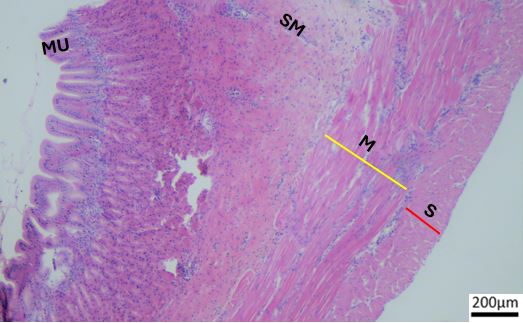

Supplement: Supplementary file 1 [file animals-14-01803-s001.zip › Supplementary file/Supplementary Figure 1.JPG]

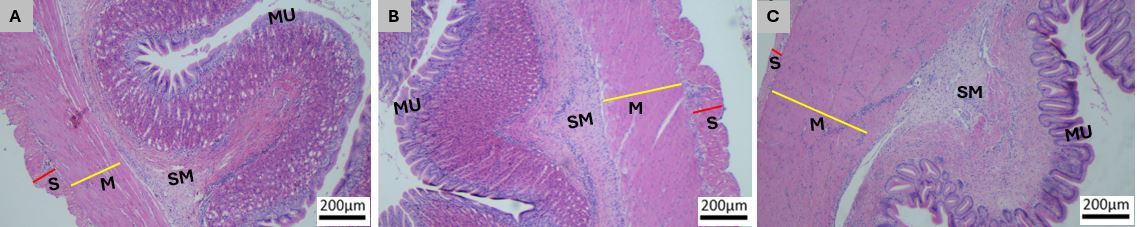

Supplement: Supplementary file 1 [file animals-14-01803-s001.zip › Supplementary file/Supplementary Figure 2.JPG]
